# Morphological Variations of the Tracheobronchial Tree in the Paediatric Population: A Systematic Review With Meta-Analysis

**DOI:** 10.7759/cureus.107074

**Published:** 2026-04-15

**Authors:** Spyridon Kanakaris, George Triantafyllou, Spyridon Prountzos, Efthimia Alexopoulou, Theano Demesticha, Dimitrios Filippou, Nikolaos Lazaridis, Maria Piagkou

**Affiliations:** 1 Department of Anatomy, School of Medicine, Faculty of Health Sciences, National and Kapodistrian University of Athens, Athens, GRC; 2 Second Department of Radiology, General University Hospital, "Attikon" National and Kapodistrian University of Athens, Athens, GRC; 3 Second Department of Radiology, General University Hospital "Attikon" National and Kapodistrian University of Athens, Athens, GRC; 4 Department of Anatomy and Surgical Anatomy, School of Medicine, Faculty of Health Sciences, Aristotle University of Thessaloniki, Thessaloniki, GRC

**Keywords:** anatomy, congenital variation, meta-analysis, paediatric anatomy, tracheobronchial tree

## Abstract

Congenital variations of the tracheobronchial tree (TBT) represent deviations from the typical bronchial branching pattern established during early embryogenesis. Although individually uncommon, these anomalies are of considerable morphological and developmental interest, particularly in the paediatric population. Therefore, the purpose of the current systematic review is to depict meta-analytic evidence for paediatric patients. A systematic literature search was conducted across major databases to identify studies reporting congenital TBT morphological variations in paediatric populations. Eligible studies were evaluated according to predefined inclusion criteria. Pooled prevalence estimates were calculated using a random-effects meta-analysis. Twenty-six studies comprising 15,734 paediatric patients were included. The most prevalent anomaly was tracheal bronchus (TB), with a pooled prevalence of 13.41%, followed by accessory cardiac bronchus (ACB) (4.03%). Less frequent anomalies included bridging bronchus (1.43%), tracheal agenesis (0.86%), tracheal trifurcation (0.40%), and bronchial agenesis (0.13%). Substantial heterogeneity and asymmetry in the small-study effect were observed in the pooled prevalence estimates. Congenital variations of the TBT are more frequently identified in the paediatric population than previously recognized. TB and ACB constitute the most common morphological variants. Accurate anatomical characterization of these anomalies enhances understanding of airway development and supports their recognition in paediatric anatomical and imaging studies.

## Introduction and background

The human respiratory system is characterized by a highly organized branching architecture that optimizes ventilation and gas exchange [1]. Morphologically, this system originates from the trachea, a median cartilaginous tube extending from the larynx into the mediastinum. At the level of the carina, the trachea divides into the right and left main bronchi [1]. The right main bronchus gives rise to three lobar bronchi, while the left main bronchus divides into two, reflecting the asymmetry of the pulmonary lobation. These lobar bronchi further branch into segmental bronchi supplying anatomically defined bronchopulmonary segments [1]. This branching pattern is established during early embryogenesis, and departures from this canonical arrangement give rise to congenital variations of the tracheobronchial tree (TBT). 

**Figure 1:**
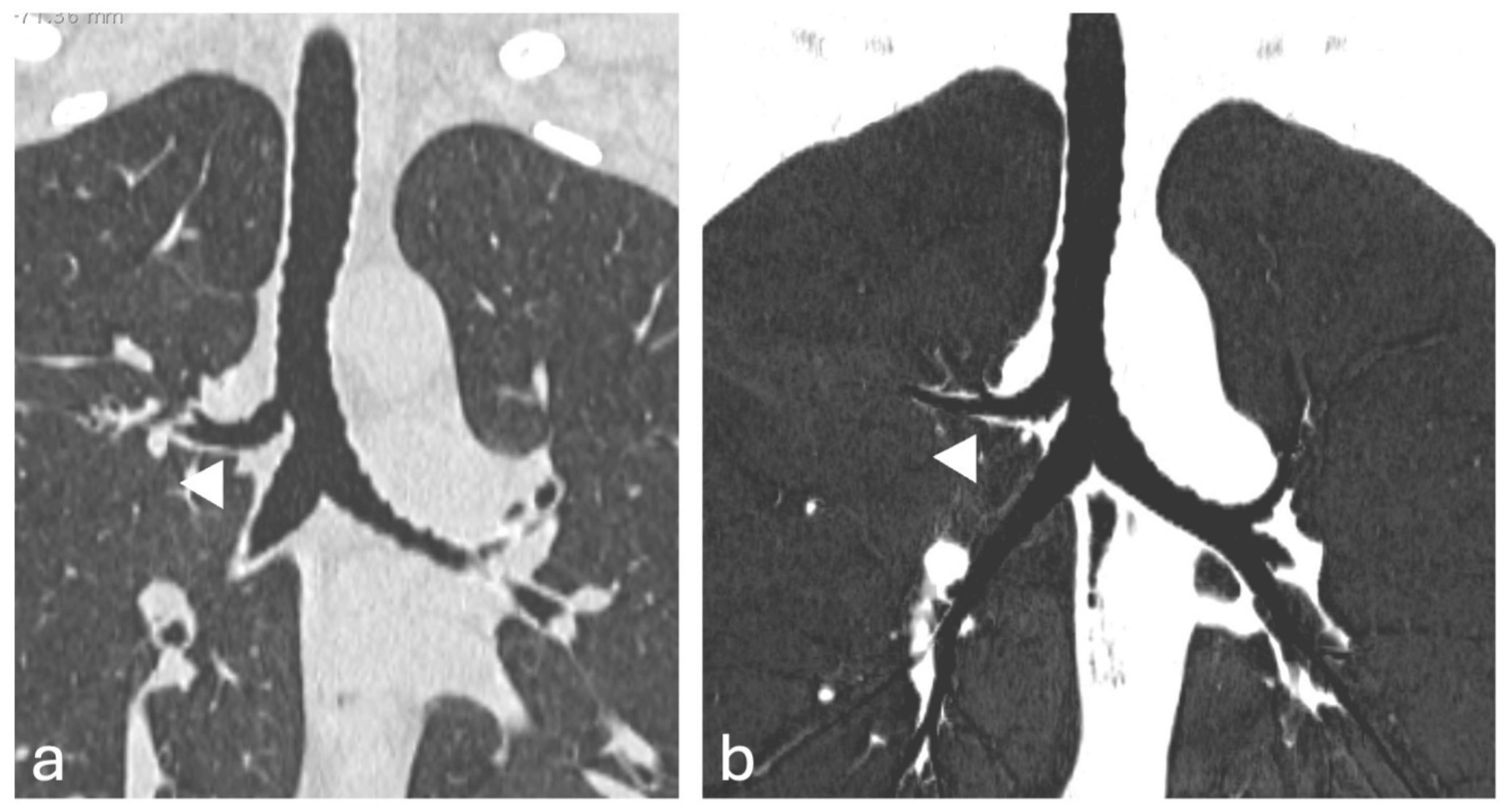
Computed tomography scan of tracheal bronchus. Coronal reformat of a chest CT scan on average (a) and minimum-intensity (b) projections depicting a tracheal bronchus (arrowheads). Image credit: Dr. Spyridon Prountzos and Prof. Efthymia Alexopoulou.

**Figure 2:**
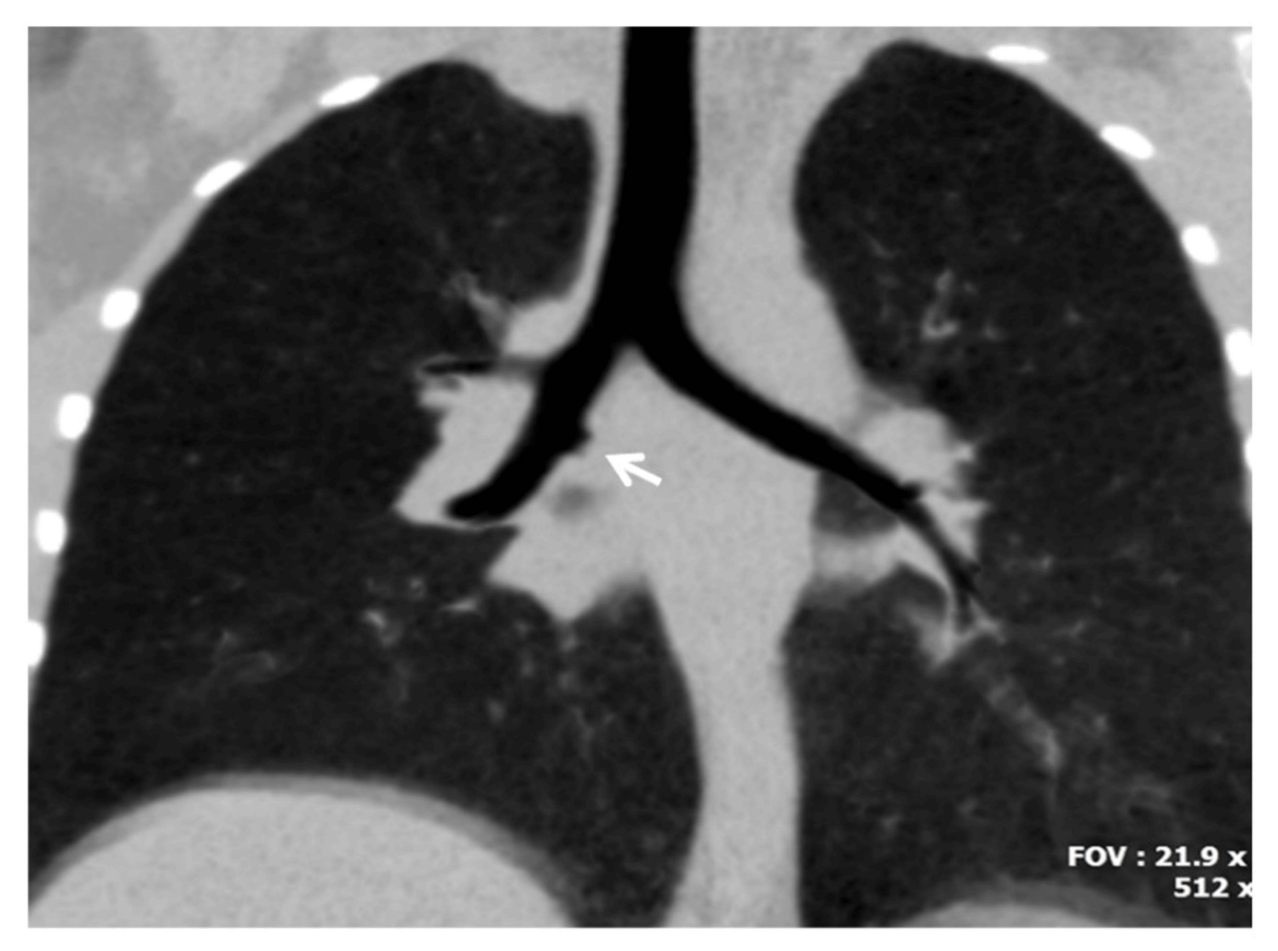
Computed tomography scan of accessory cardiac bronchus. Coronal reconstruction using minimum-intensity projection depicts a short cardiac bronchus (arrow). Image credit: Dr. Spyridon Prountzos and Prof. Efthymia Alexopoulou.

Congenital anomalies of the TBT represent uncommon morphological variants that predominantly involve proximal branching regions. Among the various described entities, the most frequently reported are the tracheal bronchus (TB) and the accessory cardiac bronchus (ACB) [2] (Figures 1, 2). These anomalies are generally attributed to aberrant budding or persistence of embryonic bronchial outgrowths during early airway development [2].

The TB is defined morphologically as an ectopic bronchial branch arising directly from the trachea, most often supplying part or all of the right upper lobe. A pooled prevalence of 0.99% has been reported in a meta-analysis [2]. In contrast, the ACB is an additional bronchial structure arising from the main or intermediate bronchus, typically projecting medially toward the pericardium and often terminating blindly. Its pooled prevalence has been estimated at 0.14% [2]. Although frequently asymptomatic, these variants constitute well-defined departures from the standard bronchial morphology.

Recent imaging-based analyses have demonstrated that the reported prevalence of TBT anomalies is higher in paediatric populations [2]. This observation likely reflects both developmental factors and improved visualization of airway anatomy using modern multidetector computed tomography (CT), which allows detailed assessment of bronchial branching patterns during early life [3]. In addition, a higher prevalence of TBT congenital anomalies has been observed in patients with congenital heart disease, supporting the concept of shared embryologic mechanisms governing airway and cardiovascular morphogenesis [3]. In light of these observations and building upon the meta-analysis by Wong et al. [2], the aim of the present study was to estimate the pooled prevalence of TBT congenital anomalies in the paediatric population using an evidence-based meta-analytic approach. Particular attention is given to the morphological characteristics of these variants and their developmental implications.

## Review

Materials and methods

This systematic review with meta-analysis adhered to the guidelines proposed by the Evidence-based Anatomy Workgroup [4] for anatomical meta-analyses and the PRISMA 2020 [5] for systematic reviews. To quantify the risk of bias, the Anatomical Quality Assurance Tool (AQUA) was used [6]. Five domains were assessed, with each one containing relevant questions with responses of “Yes," "No," or "Unclear," which assess the potential risk as “Low," "High," or "Unclear" [6].

Two independent reviewers (SK, GT) performed the database search and data extraction. The outcomes were compared, and any disagreements were resolved by the senior authors. The terms “tracheobronchial tree,” “respiratory system,” “anomalies,” “variation,” “paediatric,” “children,” “adolescence,” “anatomical study,” and “imaging study” were utilized in various combinations across the online databases PubMed, Google Scholar, Scopus, and Web of Science up to August 2025. Studies reporting the prevalence of TBT variants within the paediatric population were deemed eligible to address our research question. Studies including the adult population, case reports, conference abstracts, and letters to the editors were excluded from the current review. There were no language or date restrictions. An additional search was performed to identify further eligible articles. A manual search of grey literature and major anatomical journals (Annals of Anatomy, Clinical Anatomy, Journal of Anatomy, Anatomical Record, Surgical and Radiological Anatomy, Folia Morphology, Anatomical Science International, Anatomy and Cell Biology, Morphologie, and the European Journal of Anatomy) was conducted. Supplementary articles were also searched through the references of all included studies. The extracted data were organized into Microsoft Excel sheets (Microsoft, Redmond, WA, USA) before conducting statistical analysis.

The open-source R programming language (R Foundation for Statistical Computing, Vienna, Austria) and RStudio version 4.3.2 (Posit PBC, Boston, MA, USA) were utilized with the “meta” and “metafor” packages by a single researcher (GT). All the variables were proportions, and therefore, a prevalence meta-analysis was conducted with inverse-variance and random-effects models. The following tests and methods were applied: the Freeman-Tukey double arcsine transformation, the DerSimonian-Laird estimator for the between-study variance tau2, and the Jackson method to determine the confidence intervals (CIs) for tau2 and tau. The heterogeneity across studies was assessed through Cochran’s Q statistic, and its degree was quantified through Higgins I^2^ statistic. A p-value less than 0.10 was considered significant for Cochran’s Q statistic. According to the guidelines, the Higgins I^2^ values were considered as follows: 0-40% indicated low heterogeneity, 30-60% moderate heterogeneity, 50-90% substantial heterogeneity, and 85-100% considerable heterogeneity. To evaluate the stability and robustness of the pooled prevalence estimates, a leave-one-out sensitivity analysis was performed. This procedure involved recalculating the meta-analysis results by excluding one individual study at a time. This approach allowed for the identification of highly influential studies and served to determine if the overall results were driven by any single outlier. To evaluate the presence of small-study effects, a DOI plot with the Luis Furuya-Kanamori (LFK) index was generated [7].

Results

**Figure 3:**
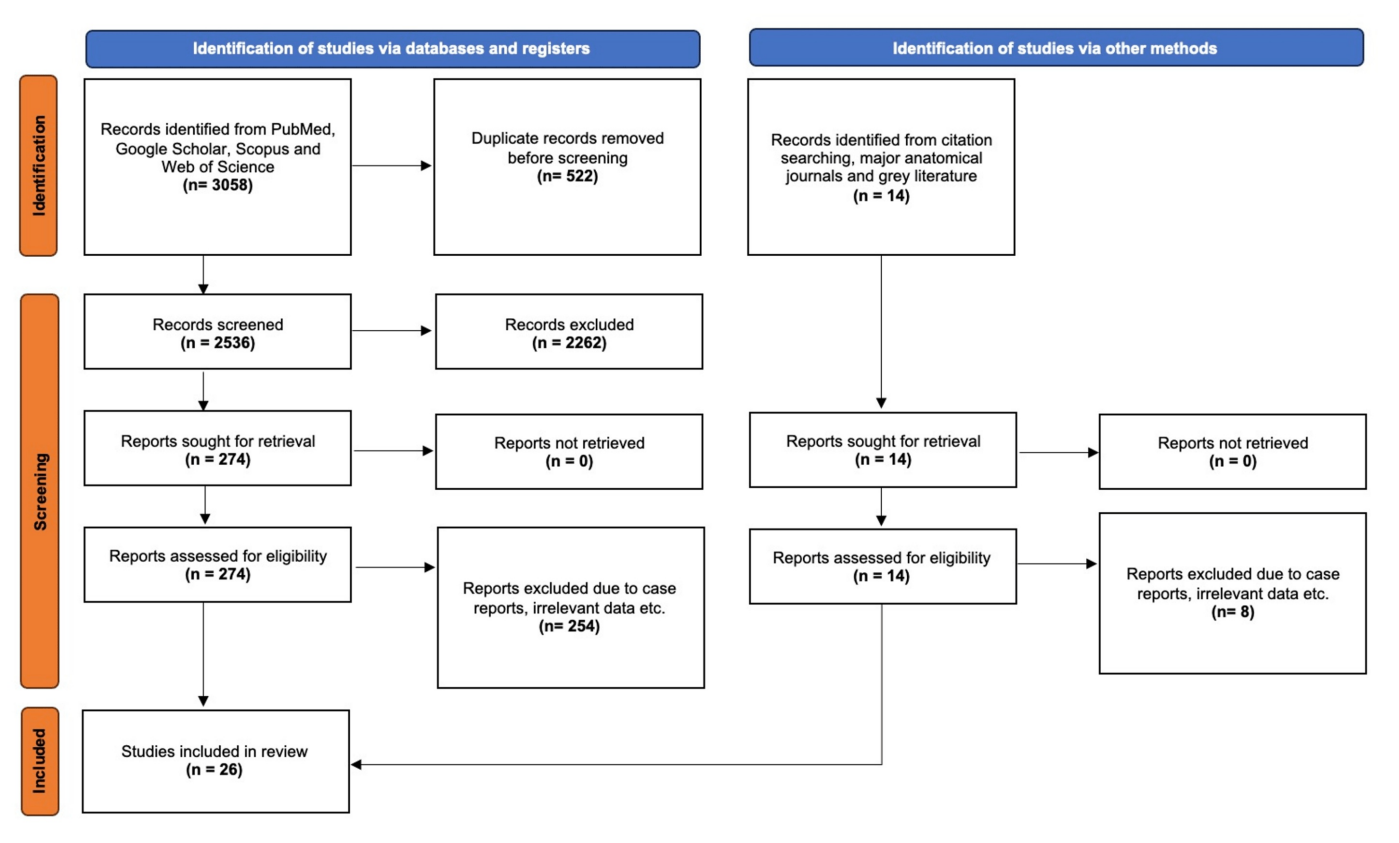
PRISMA 2020 flow chart for the systematic literature search.

The database search depicted 3,058 records, which were exported to Mendeley (version 2.10.9; Elsevier, London). Duplicated records and irrelevant articles were excluded, and 274 studies underwent full-text retrieval. The inclusion criteria for quantitative synthesis were met by 20 studies. Additionally, six studies were identified via the secondary search. In total, 26 studies were included in the present systematic review and meta-analysis. The study selection process is summarized in the PRISMA 2020 flow diagram (Figure 3) [5].

A total of 26 studies encompassing 15,734 paediatric patients were included. The publication year ranged from 1994 to 2023. Sixteen studies were based on bronchoscopy findings, nine studies were imaging-based, predominantly using CT, and one study involved cadaveric dissection. The mean sample size per study was 605.1 patients. Eleven studies were conducted in Asian populations, eight in European populations, and seven in American populations (Table 1). Nineteen studies were considered "low" risk of bias, and seven were considered "high". The detailed analysis of the AQUA tool is presented in Appendix Table 2.

**Table 1:** Characteristics of the included studies, with their risk-of-bias assessment.

Study	Year	Nationality	Type of Study	Study Design	Total Sample	Age Group	Risk of Bias
Al-Naimi [8]	2021	Asia	Bronchoscopy	Retrospective	1786	0-14 years	Low
Altman [9]	1999	America	Bronchoscopy	Retrospective	174	0-12 years	Low
Antón-Pacheco [10]	2006	Europe	Bronchoscopy	Retrospective	19	0-7 years	High
Bertrand [11]	2003	America	Bronchoscopy	Retrospective	24	0-8 years	High
Bosnali [12]	2023	Europe	Bronchoscopy	Retrospective	249	22.6 ± 22.2 months	Low
Chassagnon [3]	2017	Europe	Imaging	Retrospective	89	-	Low
Chen [13]	1994	America	Cadaveric	Retrospective	115	0-9 years	Low
Chen [14]	2001	Asia	Imaging	Retrospective	88	-	Low
Chen [15]	2003	Asia	Imaging	Retrospective	1245	0-18 years	Low
Dave [16]	2014	Europe	Bronchoscopy	Retrospective	1021	0-6 years	Low
De Lausnay [17]	2020	Europe	Bronchoscopy	Retrospective	65	0-17 years	Low
Gu [18]	2016	Asia	Imaging	Retrospective	156	0-3 years	Low
Hou [19]	2018	Asia	Imaging	Retrospective	75	0-11 years	Low
Kazim [20]	1998	America	Imaging	Retrospective	100	0-10 years	Low
Lai [21]	2013	Asia	Bronchoscopy	Retrospective	41	11.2 ± 3.0 months	High
Lee [22]	2001	America	Imaging	Retrospective	18	-	High
McLaughlin [23]	1985	America	Bronchoscopy	Retrospective	18	0-54 months	High
Moreno [24]	2019	Europe	Bronchoscopy	Retrospective	133	0-18 years	Low
Najada [25]	2011	Asia	Bronchoscopy	Retrospective	64	0-14 years	Low
Pérez Ruiz [26]	2018	Europe	Bronchoscopy	Retrospective	26	0-16 years	High
Abdul Rahim [27]	2022	Asia	Bronchoscopy	Retrospective	68	0-12 years	Low
Ruchonnet-Metrailler [28]	2015	Europe	Bronchoscopy	Prospective & Retrospective	5970	0-16 years	Low
Sánchez [29]	2003	America	Bronchoscopy	Retrospective	580	0-6 years	Low
Zheng [30]	2007	Asia	Bronchoscopy	Retrospective	396	0-6 years	Low
Zhong [31]	2010	Asia	Imaging	Retrospective	27	0-5 years	High
Zhu [32]	2007	Asia	Imaging	Retrospective	3187	0-10 years	Low

**Figure 4:**
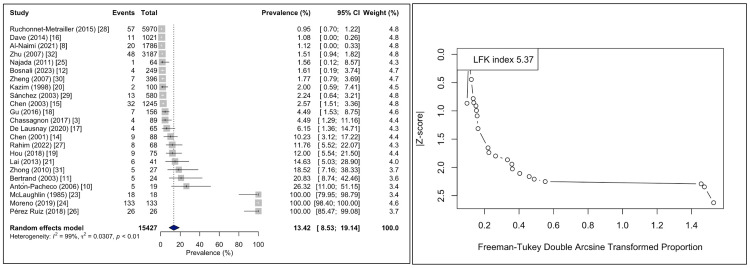
Forest and DOI plots for pooled prevalence estimates of the tracheal bronchus. The forest plot (left side) indicates the per-study prevalence and the estimated pooled prevalence, as well as the heterogeneity [3,8,10-12,14-21,23-32]. The DOI plot (right side) indicates the small-study effect based on its asymmetry that it is quantified via the LFK index. LFK index, Luis Furuya-Kanamori index.

The pooled prevalence of TB was estimated at 13.41% (95% CI: 8.53-19.14). The Higgins I² statistic was 98.5%, indicating considerable heterogeneity. The DOI plot demonstrated marked asymmetry, with an LFK index of +5.37 (Figure 4).

**Figure 5:**
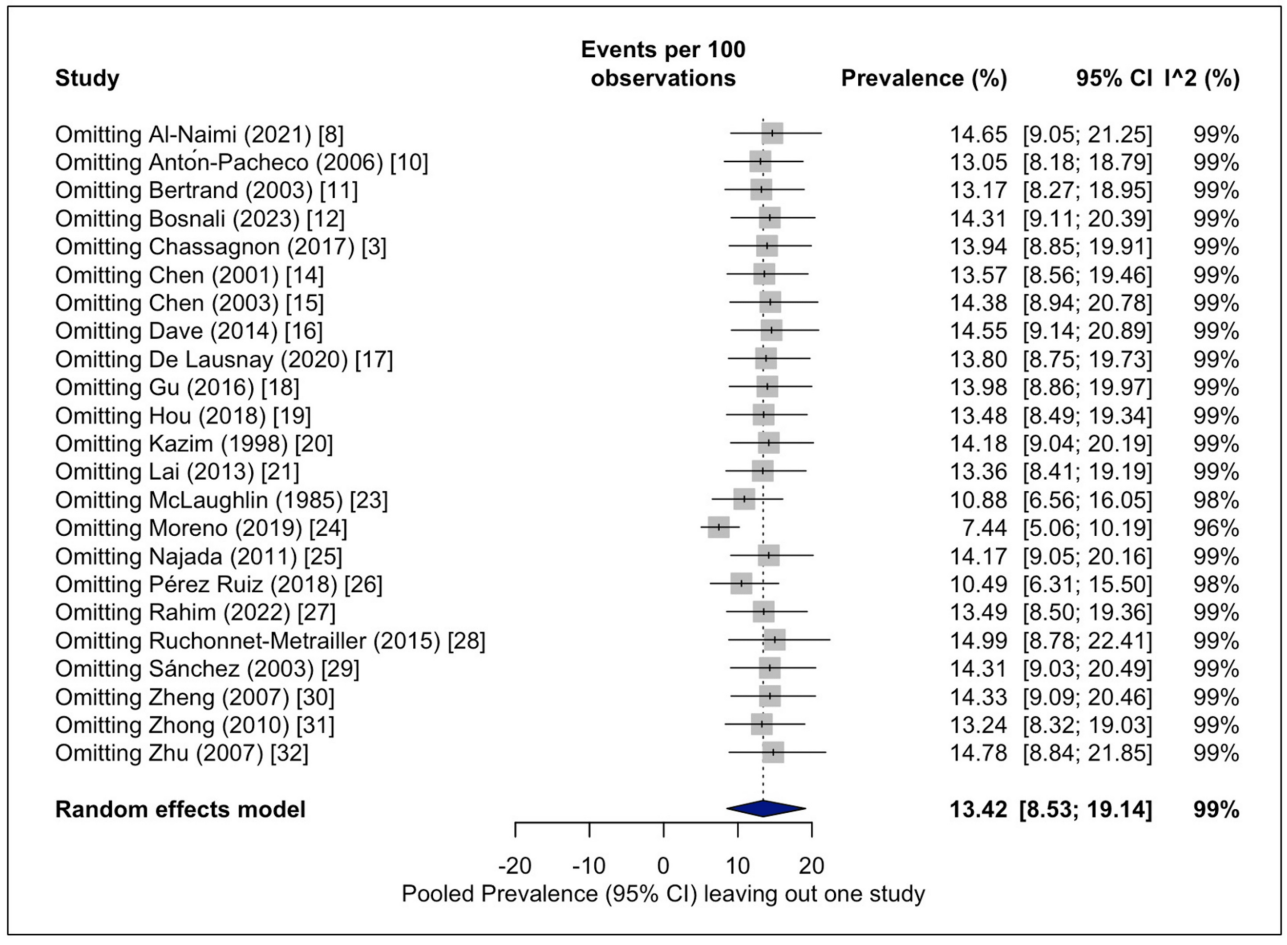
Forest plot for leave-one-out sensitivity analysis of the pooled prevalence estimates of the tracheal bronchus. The leave-one-out sensitivity analysis omitting one study per time and re-calculating the estimated pooled prevalence and heterogeneity [3,8,10-12,14-21,23-32].

The leave-one-out sensitivity analysis demonstrated that the estimate ranged from 7.4% to 14.9% and the I² statistic between 96-99%. Omitting the study by Moreno et al. [24] depicted a significant drop in the pooled prevalence (7.4%). However, the heterogeneity remained considerable (Figure 5).

Subgroup analysis by geographic region revealed a significant difference (p = 0.0085), with the highest pooled prevalence observed in American studies (26.3%) and the lowest in Asian studies (4.1%). Subgroup analysis by study type also showed a significant difference (p = 0.003), with operative studies demonstrating a higher pooled prevalence (20.1%) than imaging-based studies (4.5%).

Tracheal trifurcation demonstrated a pooled prevalence of 0.40% (95% CI: 0.02-1.07), with an I² value of 23.7%, indicating low heterogeneity. Tracheal agenesis showed a pooled prevalence of 0.86% (95% CI: 0.00-3.49), with an I² value of 47.5%, corresponding to moderate heterogeneity.

**Figure 6:**
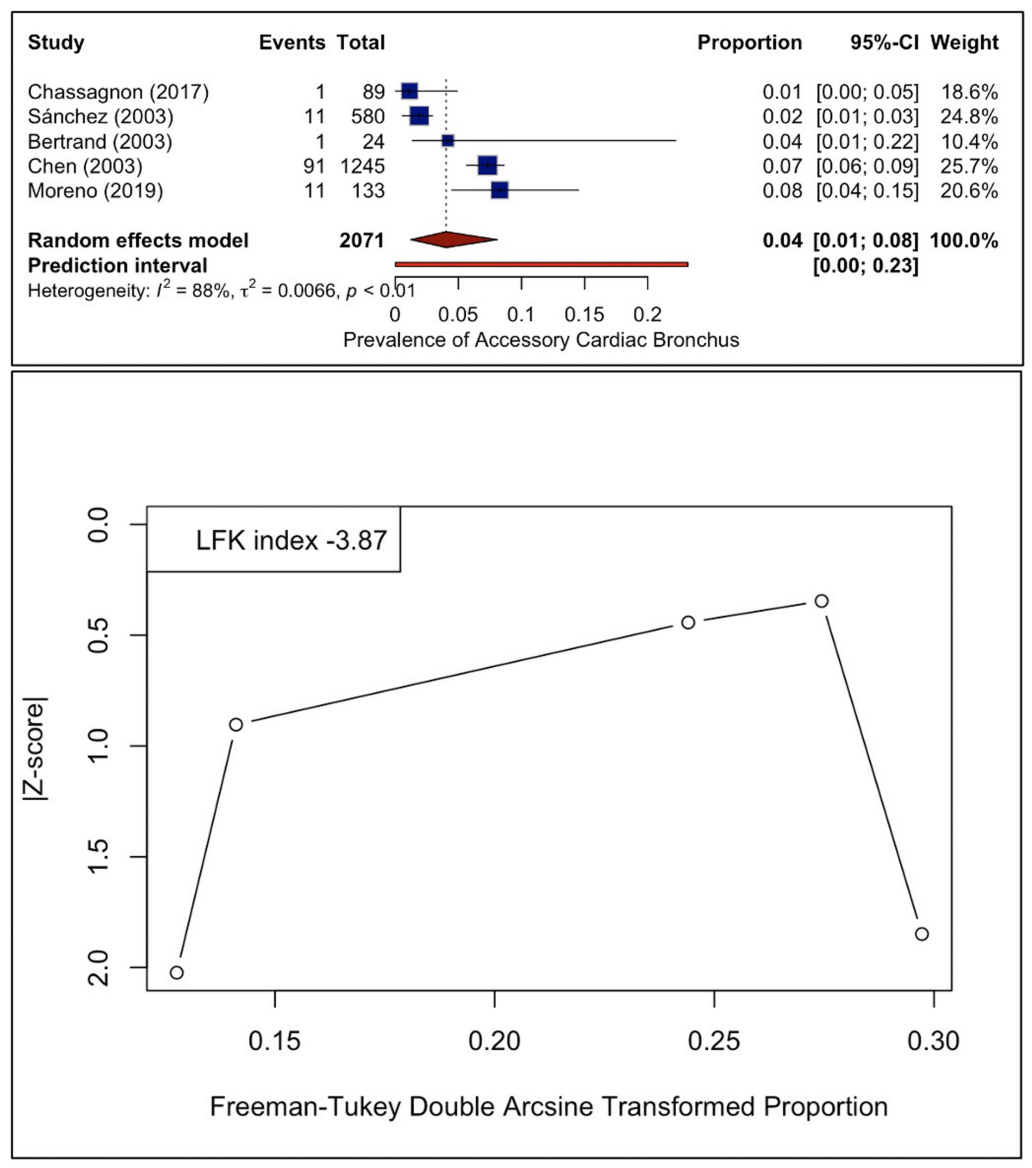
Forest and DOI plots for pooled prevalence estimates of the accessory cardiac bronchus. The forest plot (upper) depicts the per-study prevalence and the pooled prevalence estimate, as well as the heterogeneity [3,11,15,24,29]. The DOI plot (lower) depicts the small-study effect via its asymmetry that it is quantified based on the LFK index. LFK index, Luis Furuya-Kanamori index.

The pooled prevalence of ACB was 4.03% (95% CI: 1.23-8.08). The Higgins I² statistic was 88.3%, indicating considerable heterogeneity. The DOI plot revealed asymmetry with the LFK index of -3.87 (Figure 6).

**Figure 7:**
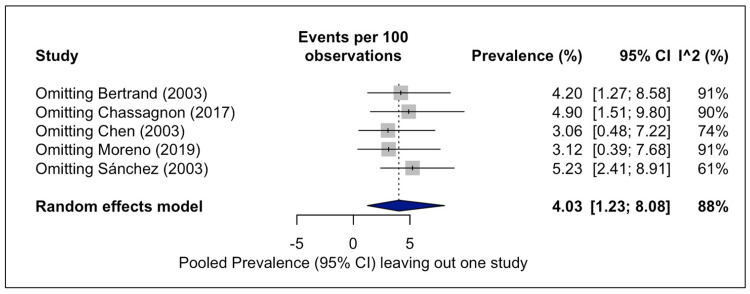
Forest plot for leave-one-out sensitivity analysis of the pooled prevalence estimates of the accessory cardiac bronchus. The leave-one-out sensitivity analysis omitting one study per time and re-calculating the estimated pooled prevalence and the heterogeneity [3,11,15,24,29].

The leave-one-out sensitivity analysis demonstrated that the estimate ranged from 3.1% to 5.2% and the I² statistic between 61% and 91%. Omitting the study by Sánchez et al. [29] significantly dropped the estimated heterogeneity to 61% (Figure 7).

Subgroup analysis by geographic region was statistically significant (p < 0.001), with Asian studies reporting the highest pooled prevalence (6.7%) and American studies the lowest (1.2%). In contrast, subgroup analysis by study type did not demonstrate a significant difference (p = 0.917).

Bridging bronchus was associated with a pooled prevalence of 1.43% (95% CI: 0.00-4.29), with an I² value of 94.4%, indicating considerable heterogeneity. Bronchial agenesis was the rarest anomaly, with a pooled prevalence of 0.13% (95% CI: 0.00-2.28) and an I² value of 92.2%, also reflecting considerable heterogeneity.

Discussion

This meta-analysis, comprising 26 studies and 15,734 paediatric patients, provides a comprehensive estimate of the prevalence of TBT congenital anomalies in childhood. Overall, the prevalence of these anomalies was substantially higher than that reported in adult cohorts, as previously summarized by Wong et al. [2]. Within the paediatric population, the most frequent variant was the TB, with a pooled prevalence of 13.41%, followed by the ACB at 4.03%. Other anomalies, including bridging bronchus (1.43%), tracheal agenesis (0.86%), tracheal trifurcation (0.40%), and bronchial agenesis (0.13%), were distinctly rarer, highlighting the wide spectrum of congenital deviations affecting proximal airway morphology.

TB represents the most frequently encountered major morphological variation of the TBT in the present meta-analysis. From an anatomical perspective, TB is defined as an aberrant or accessory bronchus originating directly from the lateral wall of the trachea above the carina, most commonly directed toward the right upper lobe [33]. The pooled prevalence of 13.41% observed in this study is markedly higher than the 0.99% reported in the adult-based meta-analysis by Wong et al. [2]. A possible explanation for this high occurrence could be that TB is associated with clinical implications (such as recurrent pneumonia) [2]; thus, these patients are increasingly investigated, leading to a higher possibility of identifying this variant.

This discrepancy can be attributed to several factors identified through subgroup and bias analyses. In particular, operative and bronchoscopic studies demonstrated a significantly higher prevalence (20.1%) compared with imaging-based studies (4.5%). As previously emphasized by Gonlugur et al. [34], bronchoscopic evaluation allows direct visualization of the tracheal wall and is therefore more sensitive to subtle morphological variants that may be overlooked on routine imaging unless dedicated multiplanar reconstructions are performed.

From a morphological standpoint, the aberrant origin and often narrowed orifice of the TB may predispose to impaired mucociliary clearance within the lung segment supplied by the TB. While TB is frequently an incidental anatomical finding in adults [35], paediatric series have consistently reported associations with recurrent focal pneumonia, chronic cough, and bronchiectasis, reflecting the functional consequences of this structural variation [35,36]. In addition, the abnormal topographic relationship between the TB and the tracheal lumen poses a recognized risk during airway instrumentation, particularly when the bronchial orifice arises high in the trachea [2,34]. These considerations underscore the importance of accurate morphological identification of TB in paediatric patients, especially in those with coexisting congenital anomalies.

Tracheal trifurcation constitutes a rare variation of carinal morphology in which three main bronchi arise simultaneously at the level of the carina. In the present meta-analysis, the pooled prevalence was estimated at 0.40%. As described by Santangelo et al. [37], this anomaly is often identified during investigations for other congenital malformations, particularly cardiovascular anomalies such as tetralogy of Fallot or pulmonary venolobar syndrome. Although tracheal trifurcation may not be inherently symptomatic, its recognition is anatomically relevant, as it alters the normal spatial organization of the carina and may complicate airway management or surgical approaches. Accurate characterization typically requires high-resolution CT with three-dimensional reconstructions, often complemented by bronchoscopy to confirm the presence and patency of all three primary branches [37].

Tracheal agenesis represents one of the most severe congenital anomalies of the airway and was identified in this meta-analysis with a pooled prevalence of 0.86%. Pfeifer et al. [38] emphasize the anatomical distinction between complete tracheal agenesis, defined by the absence of the sublaryngeal trachea, and tracheal atresia, in which a segmental remnant may be present. Embryologically, this condition reflects a failure of the primitive foregut to properly separate into respiratory and digestive components, frequently resulting in associated tracheoesophageal or bronchoesophageal fistulas [38]. From a morphological perspective, tracheal agenesis represents a fundamental disruption of airway development with profound implications for neonatal viability.

The ACB is an uncommon supernumerary bronchus arising from the medial wall of the right main bronchus or bronchus intermedius and extending caudally toward the pericardium [33,35]. In the present meta-analysis, the pooled prevalence was 4.03%, substantially exceeding the 0.14% reported in the adult-focused meta-analysis by Wong et al. [2]. As with TB, this difference likely reflects the paediatric setting, where congenital structural variations are more frequently investigated, particularly in patients with congenital heart disease (CHD) or persistent respiratory symptoms [35]. Morphologically, the ACB may terminate as a blind pouch, connect to vestigial lung tissue, or supply a small accessory pulmonary lobe [33]. Although often asymptomatic, blind-ending configurations are prone to secretion retention, which may predispose to localized infection or inflammation [35]. From an anatomical standpoint, recognition of this variant is essential to avoid misinterpretation during bronchoscopic or surgical procedures and to prevent inadvertent injury to this accessory structure.

Bridging bronchus is a rare but morphologically distinctive anomaly in which a bronchus supplying part of the right lung arises anomalously from the left main bronchus and traverses the mediastinum [39]. In this meta-analysis, the pooled prevalence was 1.43%. This anomaly is strongly associated with a left pulmonary artery sling, forming part of the so-called “ring-sling complex” [39]. The aberrant bronchial and vascular anatomy frequently results in significant airway compression and distortion of the normal tracheobronchial framework.

From an anatomical perspective, multidetector CT (MDCT) with three-dimensional reconstructions is considered essential for delineating the complex spatial relationships between the airway and vascular structures [39]. Accurate morphological characterization is critical, as these patients often require intricate surgical correction involving both the airway and cardiovascular system.

Bronchial agenesis represents a rare congenital interruption of the bronchial lumen and was identified in this study with a pooled prevalence of 0.13%. As described by Alamo et al. [40], the condition is characterized by a blind-ending bronchus with preserved distal lung parenchyma, ventilated through collateral pathways. Progressive air trapping often leads to hyperinflation of the affected lung segment, which may exert a mass effect on adjacent structures.

In the paediatric population, bronchial agenesis is frequently detected prenatally or during postnatal imaging performed for localized hyperlucency [40]. Although many cases remain asymptomatic, accurate anatomical identification is important to differentiate this entity from other congenital lung malformations, such as congenital lobar emphysema or cystic adenomatoid malformation [35,40].

Several limitations of this meta-analysis should be acknowledged. Substantial heterogeneity was observed across most pooled prevalence estimates, likely reflecting differences in study design, diagnostic modality (imaging versus operative evaluation), population characteristics, and definitions of TBT congenital anomalies across the included studies. This is also confirmed by the leave-one-out sensitivity analysis, which did not depict differences in the heterogeneity. In addition, subgroup analyses were not feasible for certain anomalies due to the limited number of eligible studies. Finally, for the rarest entities, including tracheal trifurcation, tracheal agenesis, and bronchial agenesis, the small number of reported cases restricts the precision and robustness of the corresponding prevalence estimates.

## Conclusions

This systematic review and meta-analysis provide a detailed evaluation of TBT congenital anomalies in the paediatric population. The findings demonstrate that these congenital morphological variations occur more frequently in children than previously reported in adult cohorts. TB and ACB are the most prevalent congenital anomalies, whereas bridging bronchus, tracheal trifurcation, and agenesis are distinctly rare. From a morphological perspective, recognizing and accurately characterizing these variants is essential for understanding airway development and informing diagnostic and therapeutic strategies. The routine use of high-resolution CT with multiplanar reconstructions is strongly supported for the comprehensive assessment of paediatric airway anatomy.

## Appendices

**Table 2:** The detailed Anatomical Quality Assurance (AQUA) tool of the included studies. Five domains are assessed (Objectives, Design, Methodology, Descriptive, and Results Report) and the final assessment for each study is included in Table 1. The AQUA tool was developed by Henry et al. [6].

Study	Domain 1: Objectives	Domain 2: Design	Domain 3: Methodology	Domain 4: Descriptive	Domain 5: Results Report
Al-Naimi [8]	Low	Low	Low	Low	Low
Altman [9]	Low	Low	Low	Low	Low
Antón-Pacheco [10]	Low	Low	High	High	Low
Bertrand [11]	Low	Low	High	Low	Low
Bosnali [12]	Low	Low	Low	Low	Low
Chassagnon [3]	Low	Low	Low	Low	Low
Chen [13]	Low	Low	Low	Low	Low
Chen [14]	Low	Low	Low	Low	Low
Chen [15]	Low	Low	Low	Low	Low
Dave [16]	Low	Low	Low	Low	Low
De Lausnay [17]	Low	Low	Low	Low	Low
Gu [18]	Low	Low	Low	Low	Low
Hou [19]	Low	Low	Low	Low	Low
Kazim [20]	Low	Low	Low	Low	Low
Lai [21]	Low	Low	High	Low	High
Lee [22]	High	High	Low	Low	High
McLaughlin [23]	Low	High	High	Low	Low
Moreno [24]	Low	Low	Low	Low	Low
Najada [25]	Low	Low	Low	Low	Low
Pérez Ruiz [26]	High	Low	Low	High	Low
Rahim [27]	Low	Low	Low	Low	Low
Ruchonnet-Metrailler [28]	Low	Low	Low	Low	Low
Sánchez [29]	Low	Low	Low	Low	Low
Zheng [30]	Low	Low	Low	Low	Low
Zhong [31]	High	Low	Low	Low	High
Zhu [32]	Low	Low	Low	Low	Low
